# Occurrence of filamentous fungi in maize destined for human consumption in South Africa

**DOI:** 10.1002/fsn3.561

**Published:** 2018-03-25

**Authors:** Theodora I. Ekwomadu, Ramokone E. Gopane, Mulunda Mwanza

**Affiliations:** ^1^ Department of Biological Sciences Faculty of Agriculture, Science and Technology North‐west University, Mafikeng Campus Mmabatho South Africa; ^2^ Department of Animal Health Faculty of Agriculture, Science and Technology North‐west University, Mafikeng Campus Mmabatho South Africa

**Keywords:** Filamentous fungi, human consumption, maize, South Africa

## Abstract

One‐hundred maize samples were analyzed for fungal contamination using conventional and molecular methods. The percentage incidence of different genera isolated revealed the predominance of *Fusarium* (82%), *Penicillium* (63%), and *Aspergillus* species (33%) compared to other genera. *Fusarium* occurred in 90% and 74% of small scale and commercial samples, respectively, while *Penicillium* occurred in small scale and commercial samples at an incidence rate of 64% and 62%, respectively. However, among the species, *Fusarium verticilloides* have the highest incidence of 70% and 76% in commercial and small‐scale maize, respectively, while *Penicillium digitatum* has 56% total incidence. *Aspergillus fumigatus* (27%) were also the most dominant of these genera. Fungal genera isolated included Alternaria and Cladosporium although occurring at a lower incidence level of 30%, 32% and 16%, 20%, respectively, in small‐scale and commercial samples. The results emphasize that farmers and consumers should be alerted to the danger of fungal contamination in maize.

## 1 INTRODUCTION

Microorganisms are ubiquitous in nature. They interact with plants, animals, and humans in order to ensure their growth and survival. These interactions, although in some cases are beneficial to the host, in most of the cases they are harmful interactions, causing diseases in plants, animals, and humans, as well as causing spoilage of food and feed (Basappa, 2009). Among the microorganisms, fungi, especially filamentous fungi, have assumed great economic significance. They can occur almost everywhere as they not only cause food spoilage during pre and postharvest stages of production, but may release various toxic secondary metabolites, referred to as mycotoxins (Basappa, 2009).

Maize (*Zea mays* L.) is one of the major cereals in the world and is the third highest produced grain after wheat and rice. Maize does not only serve as human food and as a feed for livestock but also an important commodity in international trade (Abassian, 2006; Basappa, 2009; O'Gara, 2007). Today, maize is directly present in the diet of more than 200 million people in different forms (Du Plessis, 2003). This consumption rate is growing annually. The FAO estimates that human and animal consumption demand for maize will be increased by nearly 300 million tons by 2030 (FAOSTAT 2012). This excludes the demand for industrial applications (O'Gara, 2007). It is also estimated that about 65% of the total world maize production is used as livestock feed, 15% as human food while the remaining 20% is mainly for industrial purposes (Abassian, 2006).

In South Africa, even though maize is the second largest crop produced after sugar cane, it is considered the most important cereal because it is a major staple diet (DAFF 2012). Maize in South Africa is consumed directly as food or is processed into different food products such as maize meal, breakfast cereals, cornflakes, grits, and snacks (Sydenham, Shephard, Theil, Marasas, & Stockenstrom, 1991). In addition, it has been estimated that the daily intake of maize in South Africa can be as high as 400 g per person (Shephard, Marasas, & Burger, 2007). The North‐west province of South Africa ranks the second major maize‐producing region in the country after the Free State. In the North‐west province, as is the case in most parts of the world, there is considerable evidence that human food especially cereal grains are frequently subjected to some form of contamination and spoilage such as fungal growth, worsened by subsequent production of secondary metabolites. Fungal growth and contamination of maize in the North‐west province is really of great concern since maize constitutes the major staple food, and is produced on a small‐scale and commercially. It is also extensively used as livestock feed and serves also as an export crop (Ncube & Flett, 2012).

With the growing awareness of food safety worldwide and also because of the various factors, including climatic changes that trigger fungal contamination of crops, threatening food security, constant evaluation for fungal contamination is needed to ensure a healthy food supply. However, molecular techniques such as pcr‐based techniques help to elucidate the difference among species based on genetic diversity. It has been shown that the Internal Transcribed Spacer (ITS) region is used to identify different fungal species (Bruns & Shefferson, 2004). The ITS region is a highly conserved priming site which makes it accessible to be amplified from virtually all fungal species. The ITS region are stretches of DNA between the 18S, 5.8S and 28S rRNA regions (White, Bruns, Lee, & Taylor, 1990). There is also the added advantage of the growing ITS sequence database that helps in the identification of various fungi. Use of this information can also allow the development of specie‐specific primers to detect certain fungi in a much shorter period than using the morphological means (Mule, Susca, Stea, & Moretti, 2004). This study was therefore conducted in other to evaluate food quality with respect to fungal contamination of maize in North‐west province of South Africa.

## 2 METHODS

### 2.1 Sampling and sample preparation

A total of 100 maize samples were collected from randomly selected small‐scale farmers (markets) and commercial farmers (silos) in the North‐west Province from April to August 2013. A total of 1 kg of each of the samples was collected in sterile plastic bags, carefully labeled and conveyed to the laboratory. Each of the samples was thoroughly mixed and milled using a sterile high speed commercial blender and packaged in sealed sterile plastic bags to avoid contamination. Samples were stored prior to analysis in the freezer.

### 2.2 Fungal culture and isolation

Fungal culture was done under aseptic conditions using dilution plating technique as described by Rabie, Lubben, Marais, and Jansen van Vuuren (1997) with the following modifications. Briefly, 1 g of the milled sample was weighed into a sterile test tube, suspended in 9 ml of sterile Ringer's solution (Sigma‐Aldrich) and vortexed. The suspension (1 ml) was serially diluted to 10^−6^ of the original concentration. One milliliter of each dilution was spread on agar plates and incubated at 30°C for 7 days. Two different media were used, Potato Dextrose Agar (PDA) and Malt Extract Agar (MEA) (Merck) and both supplemented with 80 mg/L each of Chloramphenicol and Streptomycin to suppress bacteria growth. After incubation, the fungal colonies were counted using a colony counter and the number of colonies per gram of sample was calculated and expressed in colony‐forming units per gram (cfu/g).

$$\text{Cfu}/g=\frac{\text{Number of colonies}\times \text{reciprocal of the dilution factor}}{\text{Plating volume}(1\text{ml})}$$

Subculturing of the fungal colonies was done on PDA and MEA using sterile wire loop, plates were sealed with parafilm and further incubated at 30°C for 7 days. Small portions of the pure colonies were harvested and placed on microscopic slides containing a drop of lactophenol cotton blue solution, covered with a cover slip and viewed microscopically. Macro and microscopic identification of the fungal species isolated from the samples was done according to their morphological characteristics between the 5th and 7th day of incubation using the identification keys as described by Pitt, Hocking and Klich (Pitt & Hocking, 1997) and Klich (2002). Molecular analysis was further performed in order to confirm the identity of the isolates. The isolates were further maintained by growing them on Carrot Potato Agar (CPA) slants and then stored at 4°C for further use when required. The incident rate of fungal contamination of the samples was calculated as follows:

$$\begin{array}{cc} & \text{Incident rate}\;\;(\%)\\ & =\frac{\text{Number of samples contaminated by each fungal species}\times 100}{\text{Total number of samples analysed}} \end{array}$$

### 2.3 Molecular analysis

#### 2.3.1 Fungal DNA extraction

The genomic DNA of fungi was isolated using Fungal/Bacterial DNA extraction kit (Zymo Research Corporation, Southern California, USA) according to the manufacturer's instructions. The fungal isolates were grown on PDA plates and 7‐day‐old cultures were used for DNA extraction. The mycelia were harvested by scraping the agar surface and up to about 200 mg were suspended in 750 μl of lysis solution contained in a 1.5 ml ZR Bashing Bead™ lysis tube. The eluted purified DNA was stored at −20°C until further analysis.

#### 2.3.2 Polymerase chain reaction (PCR)

The ITS region of the fungal DNA was amplified by PCR using the primer set: FF2; 5′‐GGT TCT ATT TTG TTG GTT TCT A‐3′ (forward) and FR1; 5′‐CTC TCA ATC TGT CAA TCC TTA TT‐3′ (reverse). The primer set: FF2 and FR1 was designed by Zhou et al., (2000) and produced commercially (Cruachem Inc. Aston, PA, USA). Individual reactions had 4 μl of 10–50 ng of DNA sample solution which was mixed with 25 μl master mix (Taq DNA polymerase (Fermentas Life Science, Lithuania), dNTPs, MgCl2, and reaction buffers at optimal concentrations for efficient amplification of DNA templates by PCR), 0.5 μl of each primer, that is, FF2 and FR1 and 20 μl of nuclease‐free water to make up a reaction volume of 50 μl. A negative control containing all of the reagents except the DNA was also prepared. The PCR was performed in eppendorf tubes placed in a C1000 Touch™ thermocycler (Bio‐Rad, USA). The conditions for PCR were as follows: initial denaturation of DNA at 95°C for 3 min and then 35 cycles of denaturation at 94°C for 1 min, primer reannealing at 58°C for 45 s and extension at 72°C for 1 min, 30 s. A final extension of 10 min at 72°C was included and held at 4°C until samples were retrieved. PCR products were visualized on a 2% agarose gel.

#### 2.3.3 Sequencing

PCR products were purified to remove excess primer using shrimp alkaline phosphatase and E. coli exonucleous 1. The purified products were sequenced in both directions (forward and reverse) with the primer set FF2 and FR1. Sequencing of the amplified ITS region was accomplished using the ABI PRISM^®^ 3700XL automated DNA Sequencer (Applied Biosystems, USA) at the Inqaba Biotechnological Industries (Pty) Ltd, Pretoria, South Africa.

#### 2.3.4 Species identification using DNA sequenceof the amplified ITS region

The resulting ITS region chromatograms of forward and reverse sequences of fungal DNA obtained in this study were cleaned, combined, analyzed, and edited using Chromas Lite version 2.4 software (Technelysium Pty Ltd 2012). Nucleotide sequences were analyzed and edited using the BioEdit software (Hall, 1999) to form consensus sequences. Sequence BLAST search was done and nucleotide sequences of the fungal isolates were compared with entries in nucleotide database of the NCBI web server and used to identify the specific fungi. The sequence comparison of fungal isolates showed 89%–100% identification similarities with reference species from the GenBank database.

## 3 RESULTS

### 3.1 Fungal contamination

The investigation showed that all the samples were contaminated with at least one fungal species, whereas co‐contamination with different fungi occurred in most of the samples. The percentage incidence of different genera isolated revealed the predominance of *Fusarium* (82%), *Penicillium* (63%), and *Aspergillus* species (33%) compared to other genera. *Fusarium* occurred in 90% and 74% of small scale and commercial samples, respectively, while *Penicillium* occurred in small scale and commercial samples at an incidence rate of 64% and 62%, respectively. Out of the *Aspergillus* species, 36% and 30% occurred, respectively, in small scale and commercial samples.

However, among the species, *Fusarium verticilloides* have the highest incidence of 70% and 76% in commercial and small‐scale maize, respectively, while *Penicillium digitatum* has 56% total incidence. *Aspergillus fumigatus* (27%) were also the most dominant of these genera. Fungal genera isolated included Alternaria and Cladosporium although occurring at a lower incidence level of 30%, 32% and 16%, 20%, respectively, in small‐scale and commercial samples.

## 4 DISCUSSION

The data obtained from this study (Table 1) revealed that various field and storage fungi are associated with both small‐scale and commercial maize samples. In addition, the analysis also showed contamination, predominantly by the three major toxigenic fungal spp., *Fusarium*,* Penicillium*, and *Aspergillus* in the maize samples. The findings are in line with other studies done in South Africa (Chilaka, De Kock, Phoku, Mwanza, & Augustina, 2012; Phoku et al., 2012; Shabangu, 2009) and elsewhere (Egbuta et al., 2015; Mulunda et al., 2013; Mohale, Medina, Rodríguez, Sulyok, & Magan, 2013; Pacin et al., 2002; Saleemi et al., 2012) on maize. According to Navi et al., they are the major fungal genera commonly encountered in maize‐producing regions (Navi, Bandyopadhyay, Reddy, Thakur, & Yang, 2005). *Aspergillus* and *Penicillium* spp., however, usually co‐infect with *Fusarium* spp. (Bush, Carson, Cubeta, Hagler, & Payne, 2004). In general the percentage incidence of different genera isolated revealed the predominance of *Fusarium* (82%), *Penicillium* (63%), and *Aspergillus* species (33%) compared to other genera (Table 1). This is in line with the study conducted by (Dawlal, 2010) which revealed the predominance of *Fusarium* and *Penicillium* species in South African maize cultivars. High natural contamination by fumonisin‐producing *Fusarium* species was also reported in maize from the North‐west province of South Africa (Janse van Rensburg, 2012). Jimenez, Sanchis, Santamarina, and Hernandez (1985) reported that *Penicillium* spp. are the most abundant genus after *Fusarium* spp. in corn from Spain. Dutton and Kinsey (1995) also reported *Aspergillus* spp. at lower occurrence when compared to *Penicillium* species and *Fusarium* spp.in maize.

**Table 1:** Fungal species isolated from maize grains in the North‐west Province of South Africa

Dominant spp.	Commercial		Small‐scale	
	% Incidence	Cfu/g	% Incidence	Cfu/g
*F. verticilloides*	70	1.1 × 10^6^	76	1.2 × 10^6^
*F. oxysporum*	54	3.3 × 10^4^	60	1.2 × 10^5^
*F. graminearum*	12	1.1 × 10^2^	18	1.0 × 10^2^
*F. equiseti*	16	1.5 × 10^3^	18	1.0 × 10^3^
*F. culmorum*	7	1.3 × 10^3^	15	1.5 × 10^2^
*F. solani*	4	1.3 × 10^1^	10	1.4 × 10^2^
*F. poae*	nd	nd	4	1.2 × 10^1^
*F. spp*	4	1.1 × 10^1^	9	1.0 × 10^2^
*P. digitatum*	32	1.4 × 10^2^	40	1.5 × 10^2^
*P. chrysogenum*	30	1.3 × 10^3^	36	1.1 × 10^3^
*P. spp*	6	1.3 × 10^2^	10	1.3 × 10^1^
*P. decumbens*	20	1.5 × 10^1^	18	1.3 × 103
*P. griseofulvin*	8	0.9 × 10^2^	nd	nd
*P. citrinium*	nd	nd	10	1.3 × 10^2^
*P. viridicatum*	22	1.0 × 10^2^	12	1.5 × 10^1^
*P. nalgiovince*	2	1.3 × 10^1^	4	0.9 × 10^2^
*A. fumigatus*	20	1.5 × 10^4^	22	1.0 × 10^3^
*A. candidus*	10	0.9 × 10^4^	16	1.2 × 10^4^
*A. flavus*	8	3.1 × 10^2^	12	1.5 × 10^2^
*A. ochracious*	6	1.0 × 10^3^	10	1.3 × 10^1^
*A. vesicolor*	nd	nd	6	1.3 × 10^2^
*A. spp*	4	2.3 × 10^1^	10	1.4 × 10^2^
*A. niger*	4	0.4 × 10^4^	6	1.3 × 10^2^
*Alt. alternata*	20	1.1 × 10^2^	18	1.3 × 10^3^
*Alt. spp*	10	1.5 × 10^2^	14	1.5 × 10^3^
*Cladosporium*	16	1.3 × 10^1^	20	0.9 × 104
Others	20	1.4 × 10^2^	24	1.0 × 10^2^

Cfu/g, colony‐forming unit per gram; nd, not detected; *P*,* Penicillium*;* F*,* Fusarium*;* A*,* Aspergillus*;* Alt*,* Alternaria*.

Among the species, *F. verticilloides* have the highest incidence of 70% and 76% in commercial and small‐scale maize, respectively, while *P. digitatum* has 56% total incidence. *A. fumigatus* (27%) were also the most dominant of these genera. This agrees with previous reports that *F. verticilloides* is the dominant fungal species in South African maize (Chilaka et al., 2012; Marasas, 2001; Ncube, Flett, Waalwijk, & Viljoen, 2011) as well as in maize from other parts of Africa and the world (Adetunji, Atanda, Ezekiel, & Ogara, 2014; Desjardins, 2006; Egbuta et al., 2015; van der Westhuizen et al., 2003). The predominance of *F. verticilloides* is generally associated with warm, dry climates (Fandohan, Hell, Marasas, & Wingfield, 2003; Ncube et al., 2011; Shephard, Thiel, Stockenström, & Sydenham, 1995) as is the case in North‐west province of South Africa characterized by temperatures ranging from 17 to 31°C in the summer and from 3 to 21°C in the winter. The total annual rainfall is about 360 mm with just about all of it falling in the summer months, between October and April. However, some areas of the North‐west province such as Mafikeng, Vryburg receive less rain fall and that also would explain the low‐colony forming units counted for all samples in this study.

It is important to mention that contamination of maize samples from both small scale and commercial farms by *Fusarium* spp. with the most common being *F. verticilloides*;* Fusarium graminearum*, and *Fusarium proliferatum* might be explained by the late harvesting method used in rural areas. The *Fusarium* genus is one of the major genera producing toxins such as fumonisins and trichothecenes (Pitt & Hocking, 1997). Most *Fusarium* spp. are plant pathogens and invade plant tissues and developing seeds in the fields (Mills, 1989; Pitt & Hocking, 1997), however, some are able to persist in harvested and stored grain and grow in storage when moisture content becomes favorable (Mills, 1989). This may explain their presence in these analyzed samples from both areas by late harvesting and improper storage. Dutton and Kinsey (1995), Bezuidenhout et al. (1988) and Marasas, Jaskiewics, Venter, and van Schalkwyk (1988) obtained similar results in their respective works in which fungi of the genera of *Fusarium*,* Aspergillus*, and *Penicillium* spp. were isolated from agricultural commodities and animal feeds (Bezuidenhout et al., 1988; Dutton & Kinsey, 1995; Marasas et al., 1988).

Among *Aspergillus* spp. isolated in this study, the most common ones were *A. fumigatus*,* Aspergillus candidus*,* Aspergillus flavus*,* Aspergillus ochracious*, and *Aspergillus vesicolor* (Table 1), respectively, in maize obtained from commercial and small scale farms. The presence of *Aspergillus* contamination in samples obtained from small scale farms and commercial farms might be explained by the fact that in commercial farms usually large volume of grain are produced and stored in conditions which are suitable for fungal growth, such as interactions between O_2_ and CO_2_ concentrations and moisture content (Magan, Hope, Cairns, & Aldred, 2003; Pitt & Hocking, 1997; Tuite, Koh‐Knox, Stroshine, Cantone, & Bauman, 1985) and that small‐scale farmers do not have proper storage facilities to store properly their grains. This favors humidity, fluctuating temperatures (very hot during the day and very low at night) which favor the growth of these fungi. *Aspergillus* spp. are also known to be storage fungi (Klich, 2002; Pitt & Hocking, 1997; Yiannikouris & Jouany, 2002) and they are found in corn, maize, cereals, peanuts, cotton seed, and oilseed products (Dutton, Andersson, Reiter, Razzazi‐Fazeli, & Mwanza, 2013; Klich, 2002; Yiannikouris & Jouany, 2002).

In this study the third major fungal genera which *was* isolated was the *Penicillium* genera with *P. digitatum*,* Penicillium chrysogenum*,* Penicillium decumbens*,* Penicillium griseofulvin*,* Penicillium citrinium*,* Penicillium viridicatum*, and *Penicillium nalgiovince* being the most predominant in maize collected from both commercial and small scale farmers. This presence might explain favorable environmental conditions for their growth. It is known that *Penicillium* spp. grow well between 0–30°C and have been found to produce Ochratoxin A (Pitt & Hocking, 1997). The difference in colony count and contaminations of fungi between small‐scale and commercial maize samples might be influenced by two major factors. Firstly by early (high moisture content in crops) or late (enough to favor *Fusarium* contamination) harvesting of crops in rural areas and the lack or poor storage facilities characterized by poor ventilation, high temperatures and humidity (Magan et al., 2003). Early harvesting, to avoid scavenging animals and robbers to spoil and remove crops, results in crops with high moisture content which will take longer to dry and make them prone to fungal contamination both by saprophytes such as *Aspergillus* spp. and pathogens such as *Fusarium* spp. (Pitt & Hocking, 1997; Sweeney & Dobson, 1998).

Finally among fungal genera isolated *Alternaria* and *Cladosporium* spp. were also isolated and identified although occurring at a lower incidence level of 30%, 32%, and 16%, 20% in small‐scale and commercial maize, respectively. *Alternaria* spp. are known to cause opportunistic human infections. It was also reported that the commonest clinical features are cutaneous and subcutaneous infections (Pastor & Guarro, 2008). In addition, immunosuppression is frequently associated with cutaneous and subcutaneous infections and rhinosinusitis.

Fungal growth of *F. verticilloides* suppresses relatively *F. graminearum* and *F. subglutinans* (Reid et al., 1999; Rheeder, Marasas, & Van Wyk, 1990a). This explains the low percentage incidence of *F. graminearum* of 18% and 20% in commercial and small‐scale maize, respectively, and the absence of *F. subglutinans*. Studies have also reported that maize samples with a high incidence of *F. verticillioides* are less likely to be infested with *A. flavus* and have been shown to be negatively associated with other fungal species (Rheeder et al., 1990a). This could also explain the low incidence of *A. flavus* and some other fungal isolates as reported in this study.

Small‐scale maize was mainly contaminated probably due to poor drying methods or lack of proper storage facilities, improper handling conditions, among other factors. The major cause of infection in commercial maize is mostly due to elevated temperatures in storage facilities, during transportation as well as high moisture during transportation (Mwanza, 2007). Generally, factors such as early harvesting usually result in crops with high moisture contents. This will take longer to dry making the crops prone to contamination with storage fungi such as *Penicillium* and *Aspergillus* species. While late harvesting usually favors the growth of field fungi such as *Fusarium* species some are able to persist and grow during storage when moisture content becomes favorable (Mwanza et al., 2009; Pitt & Hocking, 1997).

It is important to mention that the impacts of climate change on food safety and food security are serious issues which needs attention. Agriculture is affected by the main climatic factors (temperature, precipitation, drought, and atmospheric carbon dioxide) that are slowly but surely significantly affecting the quality of grains produced. This might also explain the presence of these fungi in the analyzed maize. A number of agricultural entities could be affected by these climatic factors, including soil quality, crop yields, and the biological environment of crops such as the abundance of pests and plant pathogens (Miraglia et al., 2009). Therefore, there is need to continuously monitor the quality of grains to assess possible climatic effects and be able to take measures before any outbreak. This is because, several genera and species of filamentous fungi produce mycotoxins that have significant agricultural, epidemiological, economic, and health impacts.

## 5 CONCLUSION AND RECOMMENDATION

This study has revealed that various fungi (field and storage) are associated with maize and previous studies have shown that at least 190 toxigenic fungi are associated with food and feed commodities in South Africa (Rabie & Marais, 2000). We were able to determine the degree of contamination and prevalence of a wide range of filamentous fungal species in maize destined for human consumption in North‐west province of South Africa. The presence of these fungal species is a reason for concern because most of the species isolated are producers of major mycotoxins such as, aflatoxins, fumonisins, ochratoxin A, zearalenone and deoxynivalenol and mycotoxin contamination is a serious food safety issue world‐wide (FAO 2004). They are known to cause numerous health effects on exposed humans and animals (Hussein & Brassel, 2001).

So, judging from the high incidence of fungal colonization observed in these grains, it is evident that consumers might be at risk of chronic mycotoxin exposure since these fungal species are known to produce a range of toxins. Hence, the presence of fungal contaminants in the samples should warrant intervention strategies to minimize their occurrence. It is therefore recommended that continuous monitoring for fungal infection, improved agronomic practices and postharvest handling of maize are necessary to minimize consequences to consumers’ health. Furthermore, disease‐management practices, such as planting of locally adapted maize varieties, reducing pest damage, early harvesting and discarding mouldy kernels should be adopted, with farmer education in rural areas which can help to reduce fungal contamination.

## CONFLICT OF INTEREST

The authors declare no conflict of interest.
